# Interactome Analysis of iPSC Secretome and Its Effect on Macrophages In Vitro

**DOI:** 10.3390/ijms22020958

**Published:** 2021-01-19

**Authors:** Luca Tamò, Kleanthis Fytianos, Fabienne Caldana, Cedric Simillion, Anis Feki, Izabela Nita, Manfred Heller, Thomas Geiser, Amiq Gazdhar

**Affiliations:** 1 Department of Pulmonary Medicine, University Hospital Bern, 3008 Bern, Switzerland; luca.tamo@dbmr.unibe.ch (L.T.); Kleanthis.Fytianos@dbmr.unibe.ch (K.F.); fabienne.caldana@anatomy.uzh.ch (F.C.); Izabela.Nita@dbmr.unibe.ch (I.N.); thomas.geiser@insel.ch (T.G.); 2 Department of BioMedical Research, University of Bern, 3008 Bern, Switzerland; Cedric.Simillion@dbmr.unibe.ch (C.S.); Manfred.heller@dbmr.unibe.ch (M.H.); 3 Graduate School, University of Bern, 3008 Bern, Switzerland; 4 Interfaculty Bioinformatics Unit, University of Bern, 3008 Bern, Switzerland; 5 Department of Gynecology and Obstetrics, Cantonal Hospital Fribourg, 1702 Fribourg, Switzerland; anis.feki@h-fr.ch

**Keywords:** lung fibrosis, macrophages, induced pluripotent stem cells, stem cells secretome, lung repair and regeneration

## Abstract

**Simple Summary:**

Macrophages play essential role in repair, regeneration and tissue remodeling. Role of macrophages in progression of lung fibrosis is established. Secretome of Induced pluripotent stem cells (iPSC-CM) has shown to reduce lung fibrosis and regulate macrophage phenotype, however exact mechanism is not known. Using advanced bioinformatics analysis by gene network analysis in this study we identified two components AAP and ELAVL-1 present in the iPSC-CM playing important role in regulation of macrophage phenotype. In this invitro study we confirmed experimentally that AAP and ELAVL1 play essential role by changing the profibrotic phenotype of the macrophages to pro resolution macrophages. We demonstrate reduction in gene expression and cytokine secretion of profibrotic macrophages after iPSC-CM treatment. Our study confirms antifibrotic and regenerative potential of iPSC-CM.

**Abstract:**

Induced pluripotent stem cell secretome (iPSC-CM) mitigate organ injury and help in repair. Macrophages play a critical role in tissue repair and regeneration and can be directed to promote tissue repair by iPSC-CM, although the exact mechanisms are not known. In the current investigative study, we evaluated the possible mechanism by which iPSC-CM regulates the phenotype and secretory pattern of macrophages in vitro. Macrophages were obtained from human peripheral blood mononuclear cells and differentiated to various subpopulations and treated with either iPSC-CM or control media in vitro. Macrophage phenotype was assessed by flow cytometry, gene expression changes by qRT PCR and secretory pattern by multiplex protein analysis. The protein and gene interaction network revealed the involvement of Amyloid precursor protein (APP) and ELAV-like protein 1 (ELAVL-1) both present in the iPSC-CM to play an important role in regulating the macrophage phenotype and their secretory pattern. This exploratory study reveals, in part, the possible mechanism and identifies two potential targets by which iPSC-CM regulate macrophages and help in repair and regeneration.

## 1. Introduction

Induced pluripotent stem cells (iPSC) offer a promising autologous patient-specific therapy option. However, its clinical translation is hampered due to safety concerns and long-term effect [1,2,3]. An alternative is the use of secretome obtained from iPSC (iPSC-CM), which contains the paracrine secreted products. The beneficial biological effect of iPSC-CM has been reported with no side effects in preclinical settings [4]. One of the initial studies reported that iPSC-CM induced alveolar epithelial repair and reduced collagen in bleomycin injured rat lung, in part due to the presence of hepatocyte growth factor (HGF) present in the iPSC-CM [5]. Another study demonstrated improvement in ventilator-induced lung injury in the rat model after iPSC-CM treatment that was mediated by interferon gamma-induced protein 10 (IP-10) present in the iPSC-CM [6]. Additionally the presence of alpha klotho in the iPSC-CM prevented hyperoxia-induced lung injury in rat lung [4]. The presence of various factors in iPSC-CM makes it a very interesting candidate to treat complex diseases where multiple pathways are involved that lack promising treatment. Idiopathic Pulmonary Fibrosis (IPF) is a complex incurable age-related disease with complicated pathophysiology [7]. Although fibroblasts [8] and alveolar epithelial cells [9] are the most widely studied cells in IPF, recent paradigms suggest that pulmonary macrophages also play a crucial role [10,11]. It has been shown that macrophages change their phenotype and secretory pattern, thus influencing disease progression [12]. Macrophages are present in all tissues and make a substantial contribution to wound healing and restoring tissue homeostasis following injury [13]. Macrophages are highly phagocytic and release various immune mediators thus modulate immune responses. Depending on their anti- or pro-inflammatory properties, macrophages are divided into two major subgroups classically activated pro inflammatory M1 and, alternatively, activated anti-inflammatory and immunomodulatory M2 macrophages. M1/M2 macrophage polarization balance governs the fate of an organ in inflammation or injury, in response to stimuli macrophages first exhibit the M1 phenotype by secreting pro-inflammatory mediators, which is followed by a switch to M2 macrophages that contribute to tissue repair and remodeling by secreting anti-inflammatory mediators [14]. M2 macrophages are further subdivided into M2a, M2b, M2c and M2d subpopulation based on their secretory profile [15]. These subgroups have different activation markers and different expression markers and have been shown to have different biological activity in vitro. The M2a macrophage is activated by IL4 or IL 13 and secretes TGFβ, IL10, CCL17, CCL18, CCL22 which have been shown to help in tissue repair by enhancing cell growth and also enhancing endocytic activity. M2b are activated by the immune complex, IL1β, and Toll like receptors, and regulate inflammatory and immune response by releasing TNFα, TGFβ, IL10 and IL6. Additionally, known as inactivated macrophages, M2c are activated by glucocorticoids and IL10 and play a critical role in the phagocytosis of apoptotic cells and secret IL-10, CCL16, CCL18 and TGFβ. The recently defined M2d subgroup is induced by the Toll-like receptor antagonist, secretes IL10 and VEGF, and helps in angiogenesis [14].

The improper activation of M2 macrophages leads to dysregulated tissue remodeling and has been reported in IPF; therefore, the restoration of M1/M2 balance is essential for healthy repair and regeneration.

Owing to the complex pathophysiology of IPF, a treatment that is dynamic and can target all involved cell types would be optimal [16]. In a previous study, iPSC-CM administration to bleomycin-injured rat lung has been shown to reduce collagen, the number of myofibroblasts and TGF-β expression in rat lung [5]. Moreover, iPSC-CM administration has also been shown to modulate the gene expression of macrophages in bleomycin injured rat lung [17]. Therefore, the complex components of iPSC-CM and their interactive modulation of multiple mediators and biological pathways might play a major role in promoting a beneficial outcome at subcellular, cellular and organ levels. In the current study, we aimed to investigate important mediators in the iPSC-CM that modulate macrophage polarization and induce lung repair. We performed in vitro experiments to test protein–protein and gene–protein interaction to identify essential components of the iPSC-CM that play pivotal role in phenotypic and secretory changes in the macrophages. We identified Amyloid precursor protein (APP) and ELAV-like protein 1 (ELAVL-1) in abundance that contributes to an alteration in the secretory pattern and gene expression of macrophages towards anti-fibrotic phenotypes in in vitro settings. 

## 2. Results

### 2.1. Cytokines Are Downregulated in M1 and Upregulated in M2a Macrophage Subpopulation Following iPSC-CM Treatment

**Figure 1:**
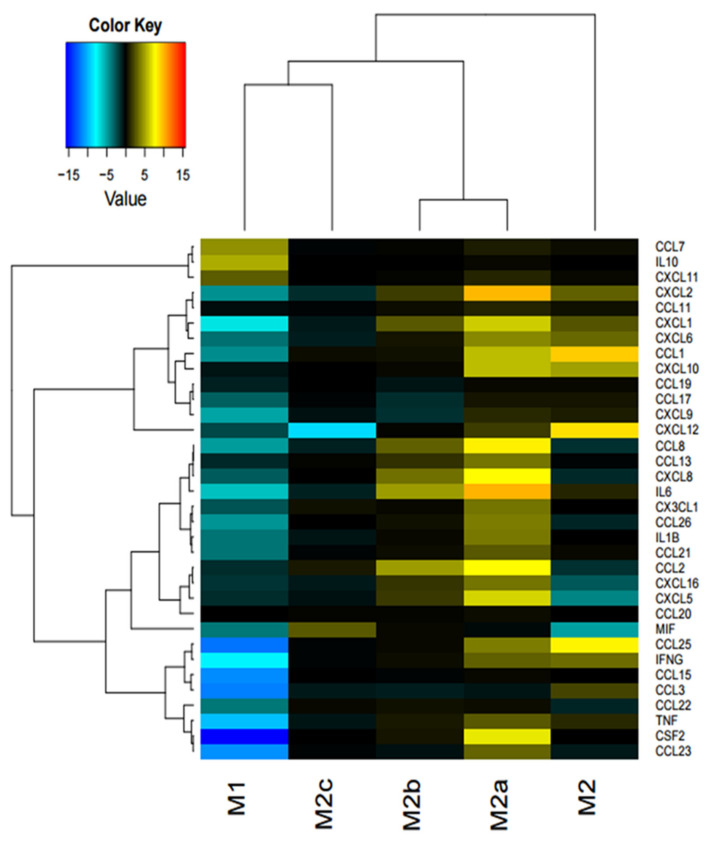
Cytokine secretory pattern was changed in M1 and M2 macrophages after iPSC-CM treatment. Heatmap shows the fold changes in cytokine secretion detected by Bioplex analysis in the different macrophage subtypes in response to iPSC-CM (*n* = 4; Mean ± SEM).

Different subpopulations of macrophages, M0, M1, M2, M2a, M2b, M2c were treated with iPSC-CM. After treatment with iPSC-CM for 24 h, cytokine secretion in M1 macrophage was reduced. In particular, CSF2 was significantly reduced, followed by CCL23, CCL25, CCL15, and CCL3. ON the contrary, in the M2 and M2a macrophages, cytokine levels were upregulated after iPSC-CM treatment. In the M2 macrophage subpopulation, CCL1, CXCL12, and CCL25 were upregulated, and IL6, CXCL2, CCL8, CXCL8, CCL2, and CSF2 were upregulated for the M2a subpopulation. The M2b subpopulation showed a trend in the upregulation of CCL8, CXCL8, IL-6, CCL2, CXCL16, CXCL5. Interestingly, however, in the M2c subpopulation, a reduction in CXCL12 only was observed, and other cytokines did not show any shift in expression (Figure 1).

### 2.2. Protein–Protein Interaction and Network Analysis Revealed Two Important Mediators Present in the iPSC-CM

**Figure 2:**
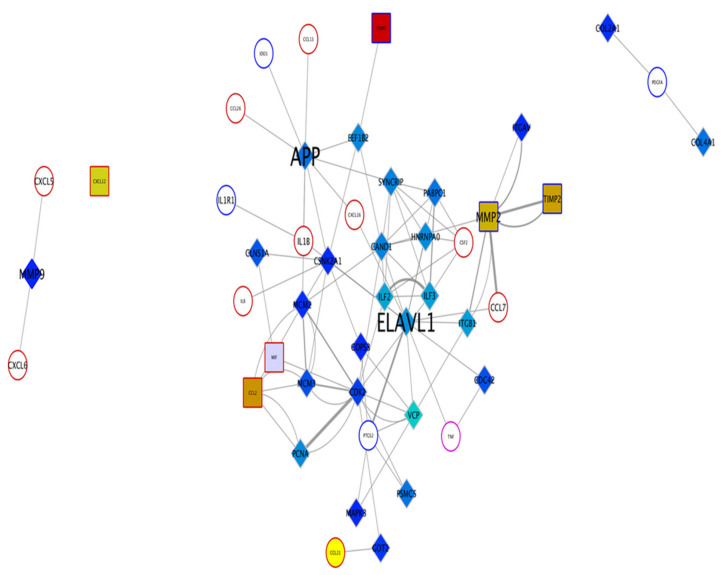
Protein–protein interaction network showing the interactions for the measured cytokines to the proteomics data obtained by the LC-MS identification of iPSC-CM proteins. Diamonds represent proteins detected uniquely in the iPSC-CM; squares represent proteins detected in both the control medium and the iPSC-CM; circles represent proteins that are measured by multiplex after treatment but were not detected in either medium. The node border color indicates the dataset; blue indicates a gene or protein present in the PCR dataset (supplementary data, Table S1 and S2); red indicates proteins that are part of the cytokine panel, and purple indicates proteins present in both datasets; grey indicates proteins that were part of neither panel. The font size indicates the degree of a node in the network.

The protein–protein interaction network revealed the Amyloid precursor protein (APP) and ELAV-like protein 1 (ELAVL-1) as two important components of the iPSC-CM that were seen interacting with more than five measured factors, either by Bioplex or RT PCR (Figure 2).

### 2.3. Depleting APP in iPSC-CM Changes Surface Marker Expression of M0 and M2c Subtype

**Figure 3:**
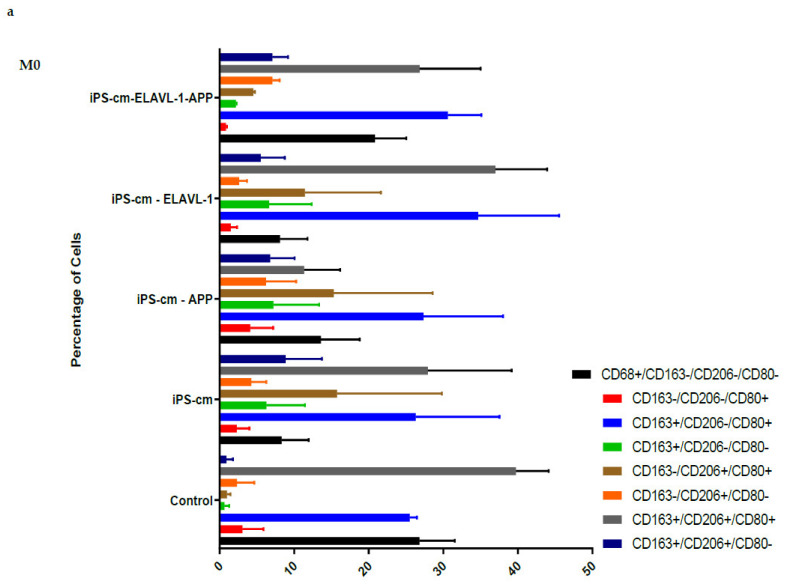

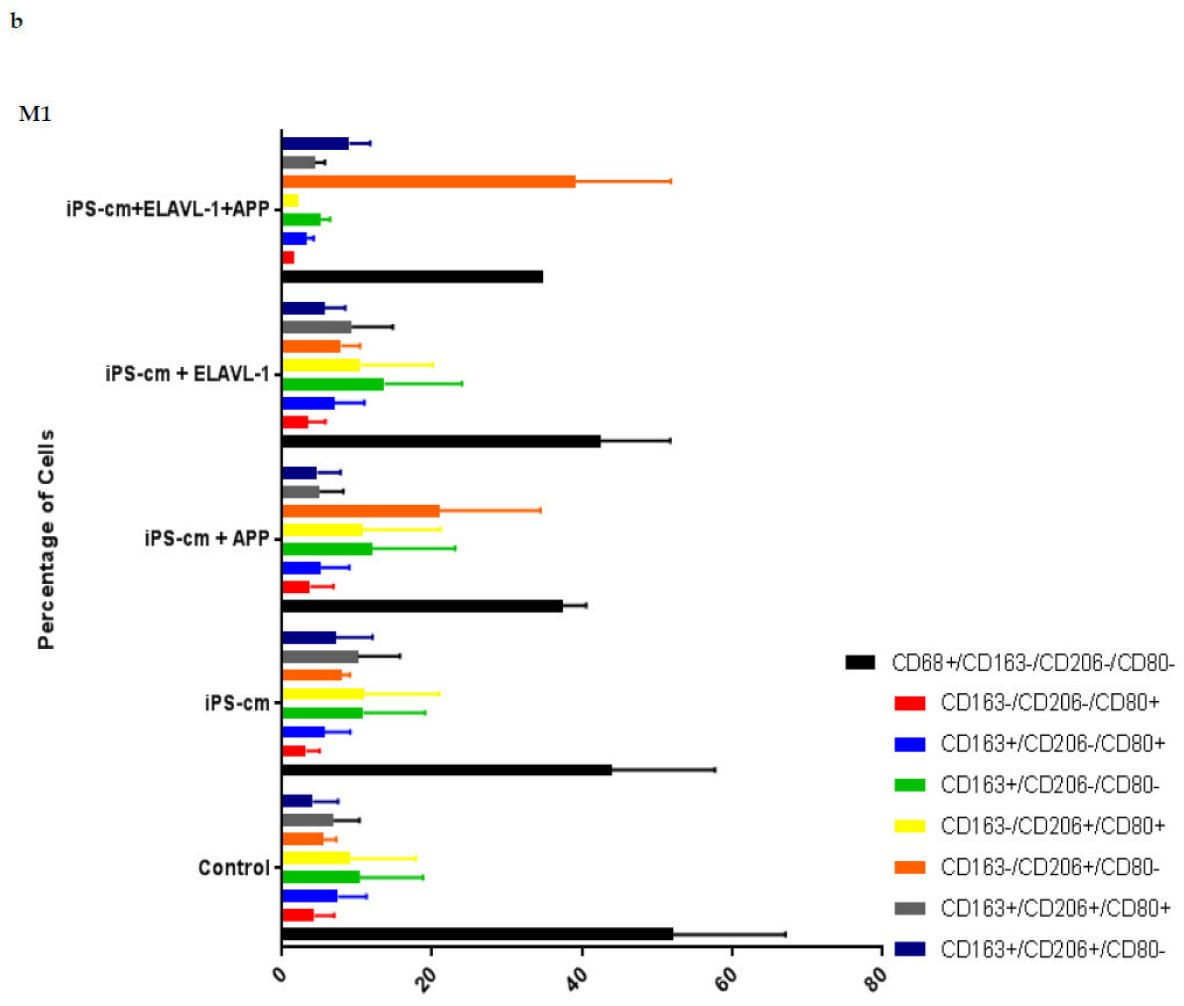

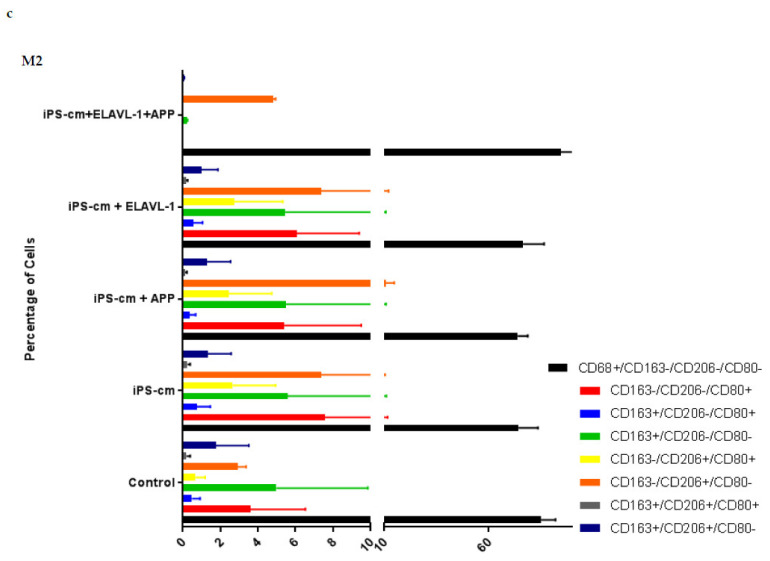

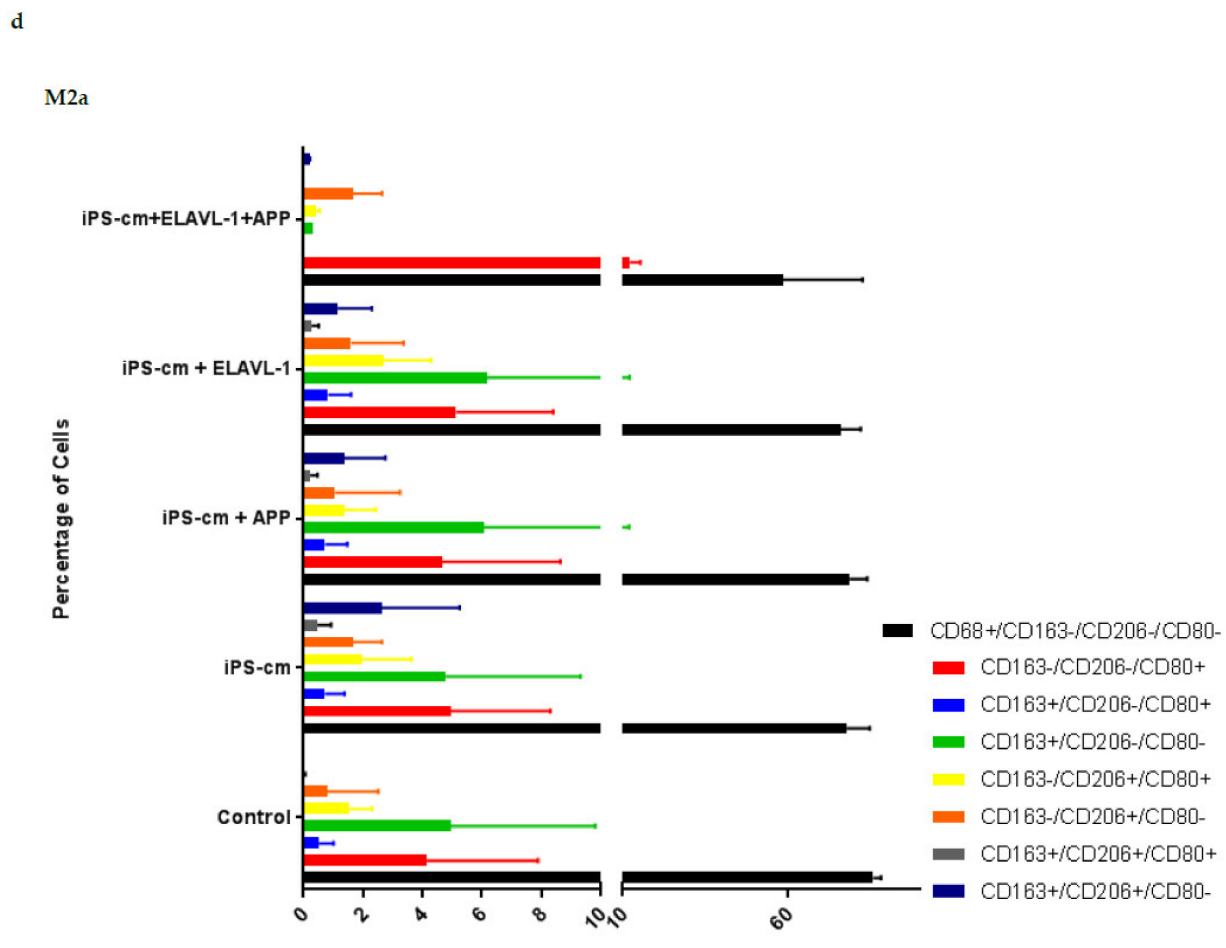

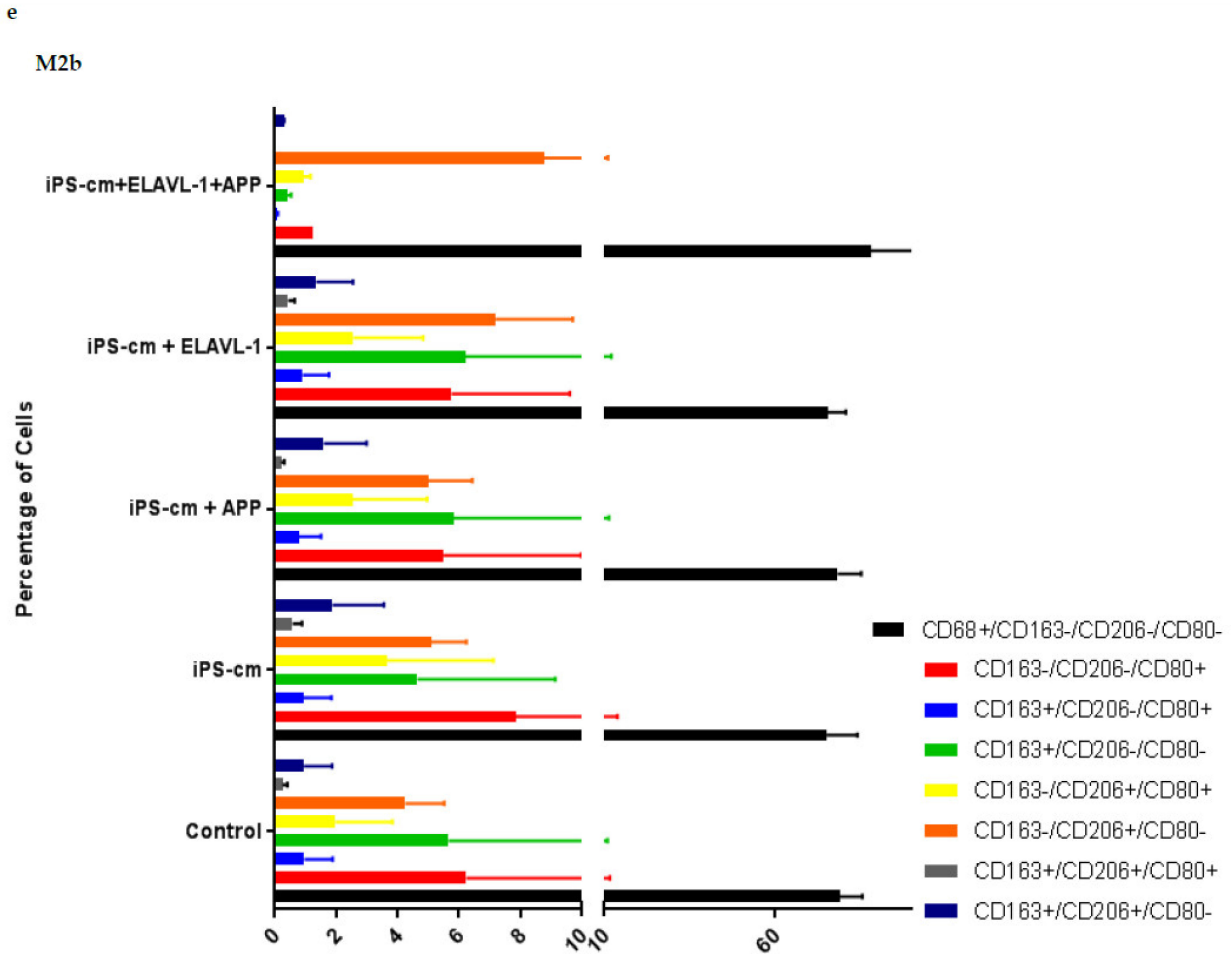

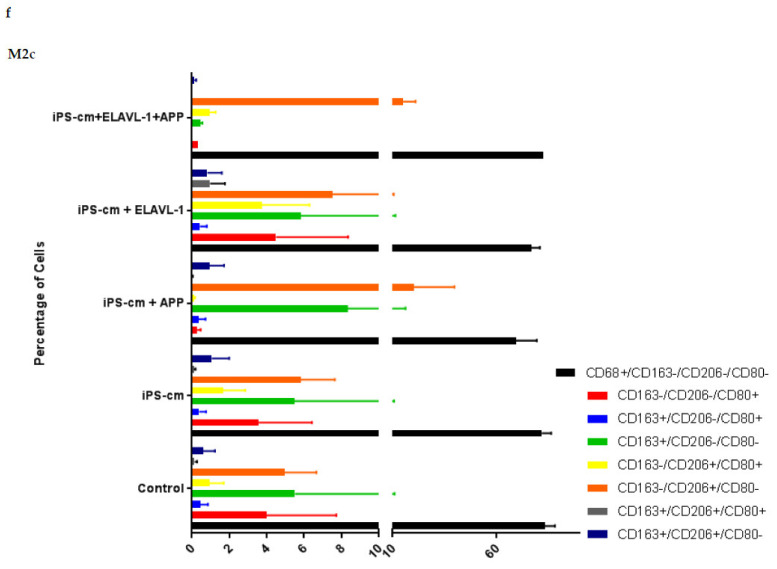
Depletion of APP altered surface expression of M0 (**a**) and M2c (**f**). Combined depletion of APP and ELAVL-1 in iPSC-CM increased CD206+ cells in M1 (**b**), M2 (**c**) and M2a (**d**) subtype. Depletion of APP in the iPSC-CM reduced CD163+/CD206+/CD80+ populations in M0 subtype compared to control. APP depletion increased CD163-/CD206+/CD80- populations in M2c subtype iPSC-CM treated macrophages (*p* < 0.05) and compared to control media (*p* < 0.05). (*n* = 4; Mean ± SEM). Finally, depleting both APP and ELAVL-1 in iPSC-CM led to increased expression of CD206 in M1, M2, and M2a subtypes. In M2c subtype, the CD206+ population was also increased, but only compared to control cells. In M2a subtype (**e**), an additional subpopulation of (CD163-/CD206+/CD80+) was increased. (*n* = 4; Mean ± SEM). Values are represented in Supplementary Materials Table S1. Flow cytometry histograms are represented as Supplementary Figures S1 and S2.

Amyloid precursor protein (APP) and ELAVL-1 appeared as important markers in iPSC-CM in the protein–protein interaction network. We investigated the effect of depleting AAP and/or ELAVL1 in iPSC-CM and tested the biological effect on each subpopulation of the macrophages. Analysis by flowcytometry revealed that depleting APP in the iPSC-CM reduced the CD163+/CD206+/CD80+ populations in the M0 subtype, compared to the control (*p* < 0.05). Depleting APP also increased the CD163-/CD206+/CD80- population in the M2c subtype, compared to the control media (*p* < 0.05) and iPSC-CM treated macrophages (*p* < 0.05) (Figure 3a,f).

### 2.4. Depleting APP and ELAVL-1 in iPSC-CM Increased CD206+ Population in M1, M2 and M2a Subtypes

Depleting both APP and ELAVL-1 in iPSC-CM led to the increased expression of CD206 in the CD163-/CD206+/CD80- subpopulations in M1, M2, and M2a macrophage subtypes. In the M2c subtype, the CD163-/CD206+/CD80- populations were also increased, but only compared to untreated cells. In the M2a subtype, an additional (CD163-/CD206+/CD80+) population was increased (Figure 3b–e) as shown by flow cytometry.

### 2.5. Effects of APP and ELAVL-1 Depletion on CCL8, CXCL10, IL-1b, MIF and CSF-2 Secretion by M2a Macrophage Subpopulation Treated with iPSC-CM

**Figure 4:**
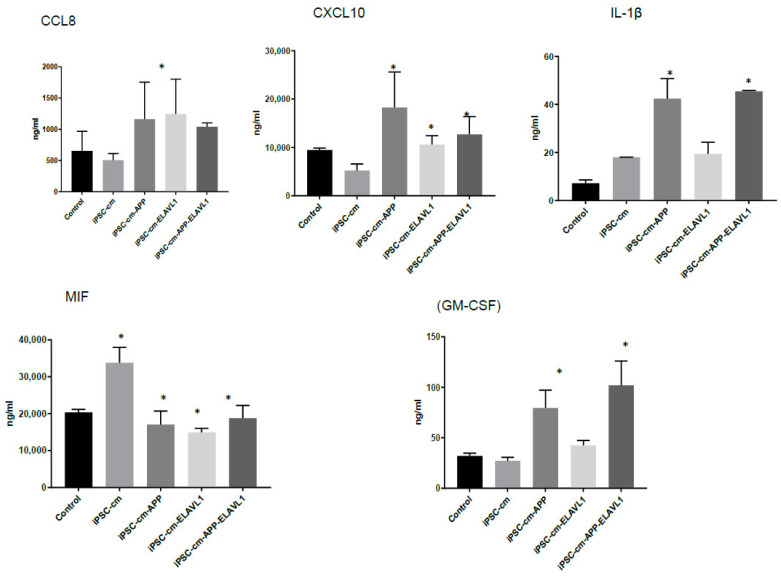
Cytokine profile of M2a macrophages after iPSC-CM treatment, as measured by Multiplex assay. Values represented as Mean ± SEM; *: *p* < 0.05.

Most significant changes in cytokine profile were observed in the M2a subpopulation. IL-1β is upregulated after blocking (APP and ELAVL-1) compared to iPSC-CM. CCL8 is upregulated after the individual blocking of APP or ELAVL-1, and also when both were blocked, compared to iPSC-CM. CSF2 is downregulated in iPSC-CM compared to the control and the levels are further reduced after single blocking APP or ELAVL-1 only, or both, compared to iPSC-CM. CXCL10 is downregulated when blocking ELAVL-1 only, and both APP and ELAVL-1 compared to the control group (Figure 4).

### 2.6. Effects of APP and ELAVL1 Depletion on PDGF, TIMP-1, PTGS2 and MRC1 Expression in M2a Macrophage Subpopulation Treated with iPSC-CM

**Figure 5:**
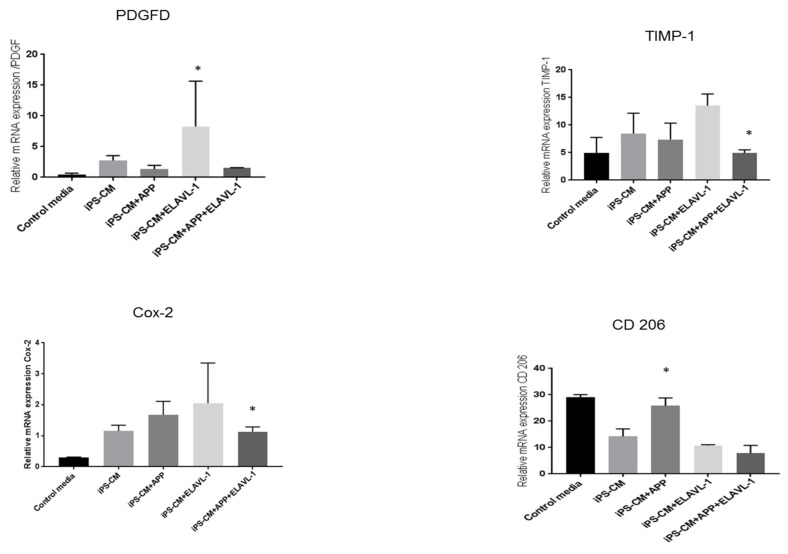
Relative mRna expression in M2a subpopulation after iPSC-CM treatment. Values are represented as Mean±SEM; *: *p* < 0.05.

The most significant effect at mRNA level was observed in the M2a macrophage subpopulation. The mRNA expression of PDGF is increased in M2a macrophages after iPSC-CM treatment, compared to the control conditions. Blocking APP alone prevented an increase in PDGF expression. By contrast, blocking ELAVL-1 did not prevent an increase in PDGF expression (Figure 5). No significant difference in TIMP-1 was observed between iPSC-CM treated and control conditions, and no effect of AAP neutralization was seen. However, TIMP-1 is upregulated after ELAVL-1 neutralization, compared to iPSC-CM (*p* < 0.05) (Figure 5). Cox-2 mRNA is upregulated in the presence of iPSC-CM. Cox-2 mRNA expression is further upregulated, although not significantly, by depleting APP. Depleting ELAVL-1 alone increased COX-2 expression. However, after depleting APP and ELAVL-1, COX-2 level is significantly reduced compared to iPSC-CM (Figure 5). Interestingly, the mRNA expression of CD206 is downregulated with iPSC-CM compared to the control; however, blocking APP increased CD206 expression (Figure 5).

## 3. Discussion

This observational study demonstrates possible mechanism by which iPSC secretome affects different macrophage subpopulations in vitro. The M2a macrophage subpopulation, in the presence of iPSC-CM, changed the profibrotic phenotype to proinflammatory phenotype. Depleting the two factors, APP and ELAVL-1, in the iPSC-CM, using specific antibodies, resulted in an increased pro-fibrotic phenotype of macrophage subpopulation, as observed by a change in surface marker, gene expression and cytokine release.

The antifibrotic properties of iPSC-CM are known [5]. In our recent study, we demonstrated that this effect is, in part, mediated by its action on macrophages in an in vivo bleomycin lung injury model [17]. Moreover, using advanced gene network analysis, we could show regulation of crucial networks involved in the fibrotic process by iPSC-CM treatment [17]. To identify specific key mediators in iPSC-CM that regulate macrophage surface expression and cytokine release, this in vitro study was performed using peripheral blood monocyte-derived macrophages. Based on protein–protein interaction network analysis of the iPSC-CM, two protiens, APP and ELAVL-1 (HuR) were chosen, since they regulated more than five cytokines released by macrophages.

Amyloid precursor protein (APP) is a protein-encoding gene associated with cerebral amyloid angiopathy and Alzheimer’s disease [18]. It has been shown to regulate neurite growth, neuronal adhesion axonogenesis and is involved in cell motility and transcription regulation [19]. Embryonic Lethal, Abnormal Vision, Drosophila (ELAVL-1 or Human antigen R (HuR)) is an RNA-binding protein associated with juvenile astrocytoma [20] and pancreas adenocarcinoma [21]. Among its related pathways are AMP-activated Protein Kinase (AMPK) signaling, and it is implicated in embryonic stem cell differentiation [22]. ELAVL-1 is known to promote proinflammatory factors in fibroblasts, T cells and macrophages [23], and has also been implicated to play a role in promoting acute respiratory distress syndrome, although the exact mechanism is still not known [24]. However, the role of APP on macrophages and lung disease has not been reported. Additionally, the combined role of APP and ELAVL-1 in chronic lung diseases and their effect on different macrophage subtypes are not known.

Monocyte-derived macrophages, when differentiated, are characterized by specific surface markers and are classified as either pro inflammatory or pro fibrotic [25]. Recently, the classification of macrophages has been revised based on their secretory pattern and surface markers expression [26]. However, in the current study for the ease of explanation and representation, we will refer to the macrophages as M1, M2 and different M2 subtypes (M2a, M2b, M2c).

We evaluated the individual and combined effects of APP and ELAVL-1 depletion in the iPSC-CM on different macrophage subpopulations. iPSC-CM treatment does not change the surface marker expression of any macrophage subpopulation compared to the control. However, by depleting both APP and ELAVL-1 in the iPSC-CM, an increase in the population that expressed only CD206 (CD80-/CD206+/CD163-) was observed in M1 and M2c macrophages. CD206 (C-type mannose receptor 1) is an M2 macrophage marker, specifically for M2a and M2c subsets [27]. Macrophages expressing CD206 are classified as profibrotic, since they promote fibroblast growth through TGF-β and CCL18 secretion [28]. Therefore, depleting both APP and ELAVL-1 in the conditioned media shifts the macrophage to a more fibrotic phenotype. Interestingly, this increase is not observed in the M2b subpopulation. Moreover, blocking both APP and ELAVL-1 resulted in an increase in CD206+ expression in M1 macrophages, indicating a shift towards a profibrotic phenotype, suggesting the possible antifibrotic role of these two proteins.

Furthermore, the down regulation of secreted cytokines, such as Colony-stimulating factor 2 (CSF2), CCL23, CCL25, CCL15, and CCL3 in the M1 phenotype was observed in response to iPSC-CM treatment. Aside from stimulating growth and the differentiation of various cells, these chemokines also have important chemotactic and chemokinetic roles. Our data show that iPSC-CM downregulates the chemotactic and chemokinetic properties of the M1 phenotype that normally recruits monocytes and macrophages to initiate the inflammatory response.

On the contrary, in response to iPSC-CM, increased cytokine levels in the M2 and M2a macrophage was observed. CCL1, CXCL12, (Stromal cell-derived factor 1 (SDF1) and CCL25 were upregulated in the M2 subset. Furthermore, for the M2a macrophage subset, IL-6, CXCL2, CCL8, CXCL8, CCL2, and CSF2 were upregulated. In summary, all the upregulated cytokines in the M2 and M2a phenotypes either recruit monocytes and macrophages, or are involved in inflammation. Interestingly, two upregulated cytokines, IL-6 and CCL2, are specific pro-inflammatory factors secreted by M1 macrophages [29]. These findings indicate that iPSC-CM influences the secretory pattern of pro-fibrotic M2 and M2a macrophage subtypes to a more pro-inflammatory subtype.

We further focused the investigation on M2a macrophages since the most interesting difference in the pattern of cytokine secretion was observed in this subpopulation in response to iPSC-CM. CCL8 was upregulated when blocking one or both factors, and IL-1β was upregulated when blocking both factors compared to iPSC-CM. CXCL10 (IP-10) was downregulated when blocking ELAVL-1 or both, compared to iPSC-CM. MIF was downregulated with iPSC-CM, compared to the control, and CSF2 was downregulated in the presence of iPSC-CM, compared to the control, and was further downregulated after the neutralization of both factors. CXCL10 is an inflammatory chemokine, and a chemotactic for monocytes and T-lymphocytes [30]. MIF is involved in inflammatory and immune responses (pro-inflammatory), in cell growth (tumor, embryo, wound healing), and has an essential role in activating T cells after mitogenic or antigenic stimuli [31]. IL-1β is a potent pro-inflammatory cytokine and produced by cells of the innate immune system. It induces the expression synthesis of Cox-2, type 2 phospholipase A, and iNOS, which leads to the production of PGE2, platelet activating factor, NO, and increases the expression of adhesion molecules [32]. The downregulated cytokines are proinflammatory and either stimulate growth and the differentiation of macrophages or recruit them. CXCL10 and IL-1β are associated with specific macrophage subtypes and both are secreted by M1 macrophages [29,33]. Interestingly CXCL10 is downregulated, while IL-1β is upregulated. Since these cytokines serve similar functions, it is difficult to speculate on the biological implications of these changes in vitro. It is clear, however, that the secretory pattern of the M2a macrophage is modified by iPSC-CM and either APP or ELAVL-1, or both play a role in regulating their secretion.

Furthermore, the mRNA expression of factors that play a role in macrophage polarization, remodeling or immunomodulation were studied in the M2a phenotype. Among the studied factors, the tissue inhibitor of Metalloproteinase 1 (TIMP-1), a remodeling factor, showed slight upregulation after iPSC-CM treatment. However, it was upregulated when ELAVL-1 alone was depleted and was downregulated when both factors were depleted, compared to iPSC-CM. TIMP-1 acts as a growth factor that regulates cell differentiation, migration and cell death. Another remodeling factor, platelet-derived growth factor (PDGF) [34] was upregulated by iPSC-CM, and was reduced by blocking APP and ELAVL-1. PDGF plays an essential role in the regulation of embryonic development, induces the influx of monocytes and macrophages and the production of the extracellular matrix [35]. These findings confirm the presence of components in the conditioned medium, which are essential for the repair processes. The mRNA expression of CD206 was downregulated with iPSC-CM. CD206 expression returned to the level of the control cells by depleting APP and ELAVL-1. Interestingly, however, there was no change in the surface marker expression of CD206 after iPSC-CM treatment or blocking, as measured by flow cytometry. Despite this variation, the data do support the hypothesis that iPSC-CM changes the pro-fibrotic macrophage phenotype to an anti-fibrotic phenotype.

Interestingly, however, the mRNA expression of COX-2 was upregulated after iPSC-CM treatment, depleting AAP or ELAVL-1, or both, which further increased COX-2 mRNA expression. Cyclooxygenase 2 (Cox-2) plays an important role in the polarization of macrophages [36]. Matrix metalloproteinase 9 (MMP9), also a remodeling factor, was downregulated with iPSC-CM, compared to the control, and was further decreased after blocking both factors. These two findings do not indicate a definite conclusion. Therefore, we speculate that this inconsistency could be either due to some intrinsic regulatory mechanisms of the macrophages or the presence of other factors in the iPSC-CM, and this needs further investigation.

The present study shows that iPSC-CM can modify secretory pattern and gene expression in macrophages, as well as surface marker expression. Furthermore, APP and ELAVL-1 play an important role in this effect. iPSC-CM has an overall positive effect in terms of ameliorating fibrosis. Future studies are warranted in order to investigate the mechanism of action of these proteins in vivo using animal models and ex vivo by testing patient material.

A limitation of the current study is that iPSC-CM comprises more than 2000 mediators. This is only an in vitro study, and we demonstrate the functional role of only two components of iPSC-CM on the macrophage subpopulation. Interestingly, APP is a membrane-protein and ELAVL-1 is nuclear protein, and their presence in the secretome might be due to the stressed culture conditions under which iPSC are cultured to collect the secretome; and this still needs to be verified. Neutralizing one or both components has yielded interesting data, indicating their essential role. However, the elaborate mechanism by which these two components act is still not known. Polyclonal antibodies were used due to the limitations of the availability of resources for blocking the two components. We used the PBMC-derived macrophages, which are not representative of alveolar or lung interstitial macrophages. Moreover, the various macrophage subpopulations in vivo are still under debate. Therefore, further, in vivo studies are required to elucidate the role of APP and ELAVL-1 in a rodent disease model to confirm our findings.

## 4. Materials and Methods

### 4.1. Generation of iPSCs and Collection of iPSC Secretome (iPSC-CM)

iPSC was generated, cultured as described before [37]. The iPSC colonies were cultured in plates coated with vitornectin media, in KO DMEM supplemented with 20% serum replacement, 1% Glutamin, 0.5% Gentamycin and 1% β-mercaptoethanol. bFGF was dissolved in the freshly made medium at a final concentration of 50ng/ml. For the collection of iPSC conditioned medium (iPSC-CM), the iPSC colonies (6.5  ±  0.53  ×  105 cells) were grown in KO DMEM, devoid of all the supplements for 24 h as described before [5]. The media was collected and centrifuged (300× *g* 5 min) to remove cell debris and stored at −20 °C until use.

### 4.2. Label-Free Protein Quantification from iPSC Conditioned Media (Proteomics)

For proteomics, both media were analyzed (a) Control media (KO DMEM media, devoid of all the supplements) (b) iPSC-CM (as described above). Proteomics was performed as described in our previous publication [4]. Conditioned cell media were incubated with 15 mL StrataClean™ resin slurry (Agilent Technologies, Basel, Switzerland) by rotation at room temperature for 60 min. Beads were sedimented by centrifugation for 2 min at 230 RCF. The medium was extracted once more. Proteins bound to StrataClean™ beads were eluted with 15 mL, reducing Lämmli buffer by boiling for 5 min. The two protein extracts were combined and the proteins separated by letting the dye front migrate only 1.5 cm into a 12.5% SDS-PAGE. Proteins were stained by Coomassie and each lane was cut into 5 horizontal slices. Each gel slice was subjected to in-gel digestion and LC-MS/MS, as described elsewhere [38] using a 40 min organic solvent gradient for peptide separation. Data acquisition on the Orbitrap XL ETD (ThermoFisher Scientific, Reinach, Switzerland) was made using a data-dependent decision tree switching between collision-induced (CID) or electron-transfer activated peptide dissociation (ETD), triggering ETD when precursor ions had a charge state of 3 or higher and the mass-over-charge values were <650 for 3+ ions, <900 (4+), <950 (5+), or no restriction with charges >5+, respectively. For ETD, the ion time for the fluoranthene reagent accumulation was set to 120 ms at an AGC target of 3e5. The reaction time was set to 90 ms and supplemental activation was used. For CID and full MS data acquisition, the parameters were as described previously [38]. CID and ETD fragment spectra were extracted to separate peak lists and searched separately against bovine and human proteins of the SwissProt protein database release 2010_12 using the CID and ETD scoring model implemented in the Phenyx search algorithm. Protein identifications were accepted, when at least 2 unique peptide sequences (at a 1% false discovery rate) were identified per protein. The peptide match score summation (PMSS) value for each identified protein was used as a semi-quantitative abundance estimate.

### 4.3. Generation and Differentiation of Macrophages

Human peripheral blood monocytes (PBMCs) were isolated from a buffy coat (Blood bank (Interregionale Blutspende SRK AG), Bern, Switzerland) and monocytes were collected by plastic adherence. The culture and differentiation of monocytes were performed using the reagents from Promocell, Germany, following the protocol provided by the manufacturer. Adherent cells were cultured with Monocyte Attachment Medium (C-28051, PromoCell, Germany), at a seeding density of 106 cells/mL, either in T75 flasks (353,136, Falcon, Cary, NC, USA) or 24-well plates (353047, Falcon, Cary, NC, USA), and incubated at 37 °C and 5% CO2 for 1 h. Cells were washed with Monocyte Attachment Medium. Depending on the desired macrophage type, 15 mL (T75 flask) or 0.5 mL (24-well plate) of either Macrophage Base Medium DXF (C-28057, PromoCell, Heidelberg, Germany), M1-Macrophage Generation Medium DXF (C-28055, PromoCell, Heidelberg, Germany), or M2-Macrophage Generation Medium DXF (C-28056, PromoCell, Heidelberg, Germany) was added. On day 7, the macrophages were activated by adding the following: human IFNγ (50ng/mL; 300-02, PeproTech, London, UK) + LPS-EB (10ng/mL; Invitrogen, Carlsbad, CA, USA) for M1 activation, human IL-4 (20 ng/mL, PeproTech, London, UK) for M2a, human IL-4 (20ng/mL) + LPS (20ng/mL) for M2b, and human IL-4 (20ng/mL) + human TGF-β1 (10ng/mL; R&D Systems, Minneapolis, MN, USA) for M2c activation. M0 and M2 did not receive any supplementary factors.

### 4.4. iPSC–CM Treatment of Macrophages

After 6 days of induction, macrophages were treated for 24 h with iPSC conditioned media iPSC-CM or iPSC-CM + anti-APP antibody (ab15272, Abcam, Cambridge, MA, USA) or iPSC-CM + anti-ELAVL-1 antibody (ab54987, Abcam, Cambridge, MA, USA) or iPSC-CM + anti-APP antibody + anti-ELAVL-1 antibody. As a control, the macrophages were grown in the media specified above. The antibodies for APP and ELAVL-1 were diluted 1:10 in the iPSC-conditioned media (0.4 μg/mL) and incubated for 30 min at room temperature. The cells were then treated either iPSC-CM or iPSC-CM with antibodies for 24 h. To test the depletion, ELISA was performed on the supernatant, and AAP and ELAVL1 were not detected in the iPSC-CM after treatment with antibodies. The experiment was repeated four times and cells from four different buffy coats were used, and each condition was analyzed in duplicate.

### 4.5. Flow Cytometry

After treatment, macrophages were collected for flow cytometry analysis. The cells were stained for macrophage surface markers to detect potential subtype alterations. The macrophage markers used were: CD68 (333809, BioLegend, San Diego, CA, USA) as a general macrophage marker, CD80 (305213, BioLegend, San Diego, CA, USA) as an M1 macrophage marker, and CD163 (555749, BD Biosciences, San Diego, CA, USA) and CD206 (321119, BioLegend, San Diego, CA, USA) as M2 macrophage markers. To exclude non-viable cells on this panel, 7-AAD marker (420403, BioLegend, San Diego, CA, USA) was used. Staining was done on ice with 30 min incubation time. At least 10,000 gated single cells were recorded. The instrument used was an LSR-II FACS from BD and the data analysis was done in FlowJo (FlowJo LLC, Ashland, OR, USA).

### 4.6. Protein Analysis Using Multiplex Assay (Bioplex)

The Bio-Plex Pro™ Human Chemokine Assay (Bio-Rad Laboratories, Herculis, CA, USA) was used to detect the concentrations of 40 cytokines (human 40-Plex kit) in the supernatant of the macrophages. The assay was performed according to the manufacturer’s protocol (Instruction manual #10031990).

### 4.7. Real-Time Quantitative PCR (qPCR)

Total RNA was extracted from the cells using the NucleoSpin^®^ RNA kit (MACHEREY-NAGEL, Dueren, Germany) following the manufacturer‘s protocol. RNA were reverse-transcribed using the Omniscript RT kit (Qiagen, Hilden, Germany), according to the manufacturer’s instructions. cDNA was amplified using Fast SYBR^®^ Green Master Mix green (Applied Biosystems, Waltham, MA, USA) and Applied Biosystems^®^ 7500 Real-Time Fast PCR (Life Technologies, Carlsbad, CA, USA). The relative mRNA expression changes were calculated using the ΔΔCt method, and the regulation factor was calculated as RF =2 − ΔΔCt. The mean quantity was normalized against the housekeeping gene18S. The list of primers is provided in Table S4 (Supplementary Materials).

### 4.8. Network Analysis and Protein–Protein Interactions

We created a protein–protein interaction network to integrate the LC-MS identification of iPSC-CM proteins, the Bioplex, and RT-qPCR results. To this end, we used the BioGRID database of protein and gene interactions. First, we retrieved all interactions between pairs of proteins identified in the iPSC-CM using LC-MS. Next, we identified all proteins in this network that interact with genes or proteins from the RT-qPCR or Bioplex data that are differentially expressed in macrophages upon treatment with iPSC-CM. Finally, we selected all proteins that are either unique to the iPSC-CM—i.e., not present in the control medium or are part of the Bioplex or RT-qPCR panels.

### 4.9. Statistics

The data were analyzed in Graphpad Prism7 (Graphpad, La Jolla, CA, USA) and a two-way ANOVA test was used. Data are shown as mean ± SEM.

## 5. Conclusions

iPSC-CM contains several beneficial mediators that act individually or synergistically and can regulate biological mechanisms that can are essential for repair and regeneration.

## Data Availability

Not applicable.

## Supplementary Materials

The following are available online at https://www.mdpi.com/1422-0067/22/2/958/s1.
