# Supplementary material for: Interactome Analysis of iPSC Secretome and Its Effect on Macrophages In Vitro
Source: Int J Mol Sci. 2021 Jan 19;22(2):958. doi: 10.3390/ijms22020958 (PMC7835982; doi:10.3390/ijms22020958)
Supplement: Supplementary file 1 [file ijms-22-00958-s001.pdf]

## Supplementary data

**Table S1:** **Frequency of macrophage surface markers:** Frequencies of macrophage surface markers (CD80, CD163 and CD206) are tabulated based on macrophage subtype and iPSCM-CM treatment. MDMs from different donors were used. Frequencies were measured from live, single, CD68+ cells in a BD LSR-II FACS. Results are expressed as Mean +/- SEM (n=3).

a)

| CD80 | M0 | M1 | M2 | M2a | M2b | M2c |
|---|---|---|---|---|---|---|
| Control | 61.0 ± 1.2 | 15.6 ± 3.0 | 7.4 ± 3.4 | 1.7 ± 0.5 | 1.7 ± 0.5 | 0.6 ± 0.1 |
| iPS-cm | 62.1 ± 8.4 | 13.9 ± 2.8 | 5.2 ± 1.5 | 1.3 ± 0.2 | 1.3 ± 0.2 | 2.1 ± 0.7 |
| iPS-cm + APP | 56.0 ± 2.8 | 7.3 ± 2.4 | 1.9 ± 1.2 | 0.8 ± 0.2 | 0.8 ± 0.2 | 0.4 ± 0.1 |
| iPS-cm + ELAVL-1 | 59.0 ± 12.2 | 20.1 ± 5.3 | 4.3 ± 1.8 | 2.6 ± 0.9 | 2.6 ± 0.9 | 4.2 ± 0.3 |
| iPS-cm + ELAVL-1 + APP | 63.1 ± 2.0 | 16.0 ± 3.2 | 3.0 ± 1.1 | 1.9 ± 0.6 | 1.9 ± 0.6 | 1.4 ± 0.2 |

b)

| CD163 | M0 | M1 | M2 | M2a | M2b | M2c |
|---|---|---|---|---|---|---|
| Control | 72.7 ± 1.2 | 27.1 ± 2.1 | 7.6 ± 0.7 | 6.4 ± 0.3 | 6.8 ± 0.3 | 7.5 ± 0.5 |
| iPS-cm | 80.1 ± 5.9 | 33.7 ± 1.1 | 7.6 ± 1.5 | 7.0 ± 0.7 | 6.8 ± 0.5 | 9.1 ± 2.6 |
| iPS-cm + APP | 75.5 ± 2.5 | 25.0 ± 1.3 | 7.8 ± 0.8 | 8.0 ± 0.4 | 8.4 ± 0.4 | 8.8 ± 0.3 |
| iPS-cm + ELAVL-1 | 81.0 ± 7.2 | 49.6 ± 5.1 | 6.4 ± 0.5 | 8.5 ± 0.8 | 8.4 ± 0.7 | 8.4 ± 0.8 |
| iPS-cm + ELAVL-1 + APP | 79.4 ± 1.0 | 51.2 ± 3.3 | 6.9 ± 0.6 | 10.3 ± 0.8 | 9.9 ± 1.2 | 9.7 ± 0.7 |

c)

| CD206 | M0 | M1 | M2 | M2a | M2b | M2c |
|---|---|---|---|---|---|---|
| **Control** | 32.9 ± 7.7 | 6.0 ± 2.3 | 7.4 ± 3.4 | 10.3 ± 1.1 | 5.1 ± 1.1 | 5.2 ± 2.0 |
| **iPS-cm** | 35.9 ± 14.1 | 18.2 ± 3.1 | 5.2 ± 1.5 | 7.8 ± 1.4 | 6.3 ± 0.9 | 6.4 ± 2.8 |
| **iPS-cm + APP** | 31.8 ± 12.1 | 52.3 ± 3.0 | 1.9 ± 1.2 | 10.2 ± 2.5 | 9.4 ± 2.8 | 36.6 ± 4.5 |
| **iPS-cm + ELAVL-1** | 52.5 ± 14.3 | 41.8 ± 17.2 | 4.3 ± 1.8 | 10.8 ± 0.7 | 9.2 ± 2.5 | 12.3 ± 3.2 |
| **iPS-cm + ELAVL-1 + APP** | 40.8 ± 4.1 | 62.1 ± 9.8 | 3.0 ± 1.1 | 38.7 ± 5.2 | 8.6 ± 1.7 | 16.5 ± 2.1 |

**Figure S1. Histograms of CD80, CD163 and CD206 expression:** % frequencies of live, single CD68+ macrophages. The effect of iPSC-cm on % frequencies is shown (upper panel).

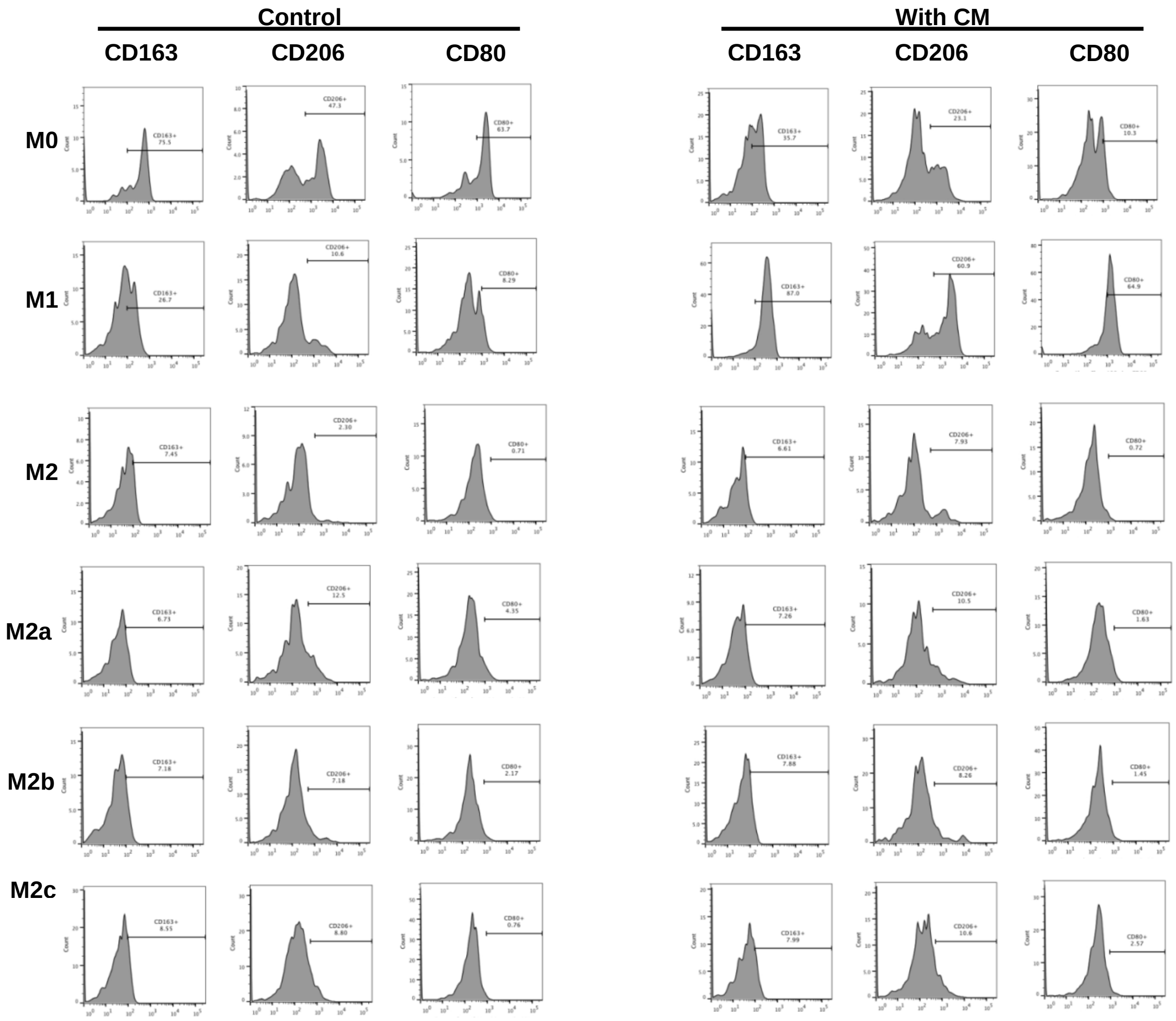

**Figure S2**. On the lower panel, the effect of APP or ELAVL1 depletion and double depletion of (APP+ELAVL1) on all macrophages subtypes treated with iPSC-cm. Frequencies were measured from live, single, CD68+ cells in a BD LSR-II FACS. A representative histogram is shown per condition (n=3).

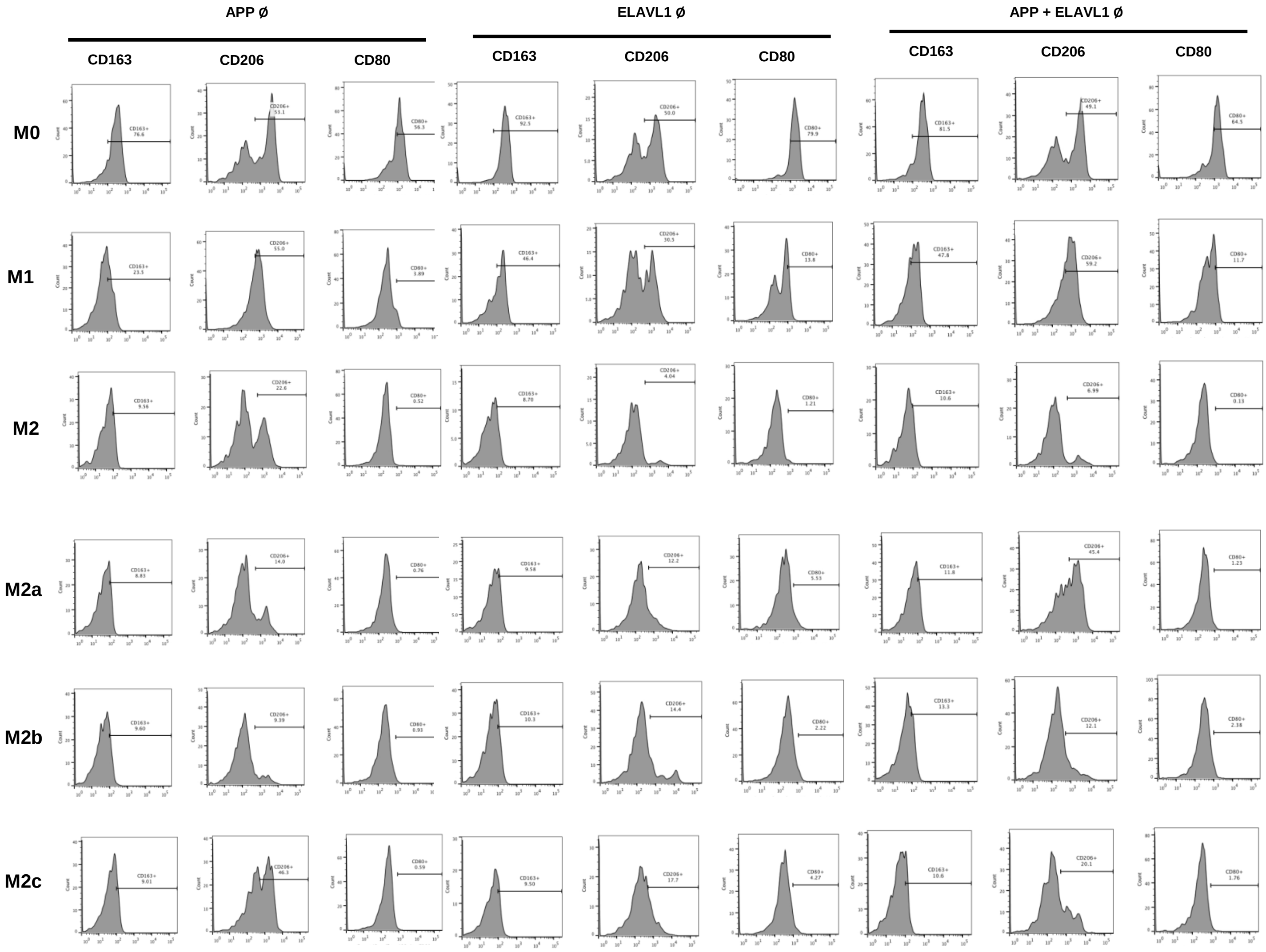

**Table S2:** Amount of cytokines secreted by M2a macrophages in response to various treatments. Values represented in ng/ml and data is presented as (mean±SEM).

| | IL-1β | CCL-8 | CSF-2 | CXCL-10 | MIF |
|---|---|---|---|---|---|
| Control | 7.2±1.4 | 653.2±316.1 | 32.04±2.67 | 9466±424.6 | 20367±805.2 |
| iPS-cm | 18.05±0.035 | 505.6±108.8 | 27.08±3.49 | 5234±1366 | 33790±4179 |
| iPS-cm + APP | 42.37±8.46 | 1164±591.2 | 79.45±17.89 | 18261±7352 | 17053±3671 |
| iPS-cm + ELAVL-1 | 19.51±4.79 | 1242±560.5 | 42.45±4.844 | 10602±1834 | 14943±1062 |
| iPS-cm+ELAVL-1+APP | 45.4±0.41 | 1040±64.58 | 102.1±23.91 | 12714±3635 | 18794±3472 |

**Table S3:** Relative mRNA gene expression in M2a macrophages in response to various treatments. Value represented as relative mRNA expression and data presented as (mean±SEM).

| | PDGFD | TIMP-1 | COX2 | CD206 |
|---|---|---|---|---|
| Control | 0.487±0.1961 | 4.927±2.83 | 0.3033±0.008819 | 29.12±0.9301 |
| iPS-cm | 2.72±0.7977 | 8.457±3.705 | 1.154±0.1861 | 14.23±2.813 |
| iPS-cm + APP | 1.332±0.6343 | 7.375±2.975 | 1.677±0.4334 | 25.83±2.986 |
| iPS-cm + ELAVL-1 | 8.293±7.37 | 13.5±2.116 | 2.053±1.296 | 10.74±0.3394 |
| iPS-cm+ELAVL-1+APP | 1.55±0.02887 | 4.911±0.5966 | 1.13±0.1559 | 7.8±3.002 |

**Table S4:** List of Primers used

| | | |
|---|---|---|
| Cox-2 | F | 5'-GTC CTG GCG CTC AGC CAT ACA G-3' |
| | R | 5'-TCC TGT CCG GGT ACA ATC GCA C-3' |
| PDGFD | F | 5'-CCT CAG GCG AGA TGA GAG CAA TCA C-3' |
| | R | 5'-TTC CTG GGG TAG CTG TTC GGG A-3' |
| CD 206 | F | 5'- TGTATTCTTTGCCTTTCCCAGTCTC-3' |
| | R | 5'-CCTCAA AACAGACTTACCCAATAGCTG -3' |
| TIMP-1 | F | 5'-GGC ATC CTG TTG TTG CTG TGG CT-3' |
| | R | 5'-TGG CCC TGA TGA CGA GGT CGG-3' |
